# Multi-Dimensional Dynamics of Human Electromagnetic Brain Activity

**DOI:** 10.3389/fnhum.2015.00713

**Published:** 2016-01-19

**Authors:** Tetsuo Kida, Emi Tanaka, Ryusuke Kakigi

**Affiliations:** Department of Integrative Physiology, National Institute for Physiological SciencesOkazaki, Japan

**Keywords:** inter-regional connectivity, graph theory, complex systems, non-linear dynamical system, neuronal avalanche, self-organized criticality

## Abstract

Magnetoencephalography (MEG) and electroencephalography (EEG) are invaluable neuroscientific tools for unveiling human neural dynamics in three dimensions (space, time, and frequency), which are associated with a wide variety of perceptions, cognition, and actions. MEG/EEG also provides different categories of neuronal indices including activity magnitude, connectivity, and network properties along the three dimensions. In the last 20 years, interest has increased in inter-regional connectivity and complex network properties assessed by various sophisticated scientific analyses. We herein review the definition, computation, short history, and pros and cons of connectivity and complex network (graph-theory) analyses applied to MEG/EEG signals. We briefly describe recent developments in source reconstruction algorithms essential for source-space connectivity and network analyses. Furthermore, we discuss a relatively novel approach used in MEG/EEG studies to examine the complex dynamics represented by human brain activity. The correct and effective use of these neuronal metrics provides a new insight into the multi-dimensional dynamics of the neural representations of various functions in the complex human brain.

## Introduction

Neuroscience has been witness to a long-standing debate on functional specialization (or localization) of the human brain since the early times of Franz Josef Gall, Marie-Jean-Pierre Flourens, Paul Broca, John Hughlings Jackson, and other individuals of note in this field. Pioneering research, which examined single-neuronal activity using micro-electrodes in conscious monkeys (Evarts, 1968; Hubel and Wiesel, 1968; Bruce et al., 1981) and human patients (Quiroga et al., 2005), revealed that neuronal activity patterns differed depending on brain regions, thereby providing evidence for functional specialization. Recordings of electric potentials from several regions of the brain (Allison et al., 1991, 1999) and their stimulation in humans and animals (Penfield and Rasmussen, 1950; Parvizi et al., 2012) further supported functional specialization. Functional magnetic resonance imaging (fMRI) (Ogawa et al., 1990; Bandettini et al., 1992; Friston et al., 1994) has also markedly accelerated the concept of functional specialization. Previous studies have identified a large body of representations in the brain, e.g., for retinotopy (Sereno et al., 1995; Wandell et al., 2007), tonotopy (Langers et al., 2007), somatotopy (Martuzzi et al., 2014), multisensations (Downar et al., 2000; Beauchamp et al., 2004), the face (Puce et al., 1996; Kanwisher et al., 1997), body parts (Downing et al., 2001), mirror system (Iacoboni et al., 2005), the theory of the mind (Gallagher et al., 2000), and even love (Bartels and Zeki, 2004), in association with fMRI studies on non-human primates (Tsao et al., 2006, 2008). Furthermore, lesion studies on human patients (Vaina, 1989; Damasio et al., 1994) and activation studies using positron emission tomography (PET) (Roland et al., 1980; Corbetta et al., 1990) have also provided essential evidence for functional specialization. Magnetoencephalography (MEG) has also demonstrated functional specialization in sensory cortices by estimating somatotopy (Baumgartner et al., 1991), tonotopy (Romani et al., 1982; Pantev et al., 1989), and retinotopy (Aine et al., 1996; Supek et al., 1999). Thus, the idea of functional specialization in non-invasive neuroimaging has been examined extensively and is now largely accepted. In these fMRI, PET, MEG, and EEG studies, the spatial patterns of differences in activation between a task and the baseline, and between different tasks or stimuli have been one of the most common sources of evidence for functional specialization. We herein refer to this type of activity difference as the first category of neural indices. Virtual lesion studies using transcranial magnetic stimulation (TMS) have been another crucial source for functional specialization.

Individual brain regions have billions of reciprocal structural connections, and send neuronal signals to each other in the form of action potentials. This type of reciprocal information transfer may directly or indirectly change temporal patterns or phase differences between population-level neural signals recorded from different brain regions using neuroimaging techniques. Therefore, unlike the functional separation of brain regions, signal transmission or information transfer between brain regions cannot be assessed based only on differences in the magnitude of population-level neuronal or hemodynamic activation. Inter-regional connectivity may have the capacity to estimate cortical information transfer, and is computed from structural and anatomical brain signals in various manners. Connectivity includes structural and functional connectivities, which are obtained by analyzing anatomical MR signals and functional neuroimaging signals, respectively. Structural or anatomical connectivity is obtained using diffusion spectral imaging (DSI) (Hagmann et al., 2007, 2008), diffusion tensor imaging (DTI), and cortical thickness (Iturria-Medina et al., 2007), whereas functional connectivity was initially defined as temporal correlations between spatially remote neurophysiological events in an early PET study (Friston et al., 1993b; Friston, 1994). Thus, functional connectivity itself provides no directed information, i.e., no information on the causality of a relationship, whereas effective connectivity typically comprises an estimate of directed influence between brain regions (Friston et al., 1993a). These concepts originated from multiunit electrode recordings (Gerstein and Perkel, 1969; Gerstein et al., 1989; Aertsen and Preissl, 1991; Gochin et al., 1991). Many metrics for functional and effective connectivities are currently computed from brain signals measured using various sophisticated recording techniques including fMRI, electroencephalography (EEG), MEG, near-infrared spectroscopy (NIRS), electrocorticography (ECoG), and local field potential (LFP). We herein defined connectivity as the second category, and focused on MEG/EEG connectivity.

The third category is based on an advanced and integrated view of connectivity, and is computed using a complex network or graph-theoretical analysis (Watts and Strogatz, 1998; Barabasi and Albert, 1999; Bullmore and Sporns, 2009; Sporns and Bullmore, 2014; Stam, 2014). Individual brain regions or neurons (nodes or vertices) are activated through neuronal connections (links or edges), and function locally and globally as a network to realize complex human perceptions, cognition, and actions. Connectivity is the strength or degree of the relationship between signals from two or more brain regions (or neurons), while graph metrics describe the various local and global properties of a network by taking many connectivity values into consideration. These three categories of neuronal metrics derived from multichannel, rapidly time-varying brain signals provide complementary information on each other. In recent years, marked advances have been made in the latter two categories, connectivity and graph metrics, which we herein review. The connectivity and graph-theoretical analyses also lead to a rediscovery and extension of the idea of functional specialization in terms of network and its elements. We will briefly describe the basics of MEG/EEG, followed by an overview of connectivity and graph metrics. We will then provide a short overview on another recent topic in human neuroscience regarding dynamical and complex systems, which has been attracting interest. Experts on MEG/EEG may skip the next section “Basics of MEG/EEG” and proceed to the sections “Connectivity” and “Network metrics.”

## Basics of MEG/EEG

MEG is an invaluable functional brain imaging technique that provides direct, real-time monitoring of neuronal activity with high temporal resolution (Cohen, 1968, 1972; Hämäläinen et al., 1993; Kakigi et al., 2000; Supek and Aine, 2014; Hari and Parkkonen, 2015). The cerebral cortex in the human brain is a 2–4-mm-thick sheet of gray matter that generally has 5 or 6 layers. Pyramidal neurons are aligned in the same direction perpendicularly along the surface of the cortex, in which there are at least 10^10^ neurons. When large numbers of neurons with the same orientation in restricted cortical layers are synchronously activated by trans-synaptic inputs, it is possible to detect the resulting magnetic fields using magnetic field sensors placed near the scalp. These trans-synaptic inputs induce a primary current, which is related to the movement of ions due to their chemical concentration gradients, and passive ohmic current (also called volume current), which occurs in the surrounding medium, in the brain as a volume conductor, with the latter current completing the loop of ionic flow in order to prevent the buildup of charge in the conductor (Hämäläinen et al., 1993). Although magnetic fields are generated from primary and volume currents in the brain, the volume current does not produce any magnetic fields outside a symmetric volume conductor, and, thus, contributes less to the extracranially-recorded magnetic field. Radial current sources also do not produce any magnetic fields outside a symmetric volume conductor, whereas tangential current sources do. Therefore, MEG is advantageous for recording activity from the cortical sulci, but may also measure magnetic fields originating from cortical gyri to a certain extent because no cortices are oriented completely perpendicular to the scalp or brain surface (to the surface of sensors) (Hillebrand and Barnes, 2002; Ahlfors et al., 2010). EEG records the time series of a potential difference (voltage) between two sites (recording and reference sites). EEG signals mainly include volume currents originating from both radial and tangential current sources. EEG is a classical technique that has been used for approximately 100 years since its first application to humans (Berger, 1929). Recent advances in multichannel recordings, signal processing, portability, multimodal recordings, and multi-person recordings have rendered EEG a unique and powerful neuroimaging tool. Although the spatial resolution of EEG is sometimes regarded as being inferior to that of MEG, proper sampling and the correct analysis of electric fields provide reliable information on neuronal activity with high temporal resolution (Michel and Murray, 2012).

The most important advantage of MEG/EEG is excellent temporal resolution in the order of milliseconds. Temporal resolution may be considered in two manners; one is as an aspect of the measuring device, while the other is as a physiological aspect depending on its electromagnetic properties and the temporal profile of underlying neuronal activity. This advantage also enables examinations in a wide range of frequencies from near-DC to more than 100 Hz by transforming time-dimension data to frequency-dimension data with, for example, Fourier, Wavelet, Stockwell and Hilbert analyses, which provides two-dimensional neural representations in time and frequency in different brain regions (time-frequency representation, TFR). Moreover, it is possible to consider a three-dimensional representation by adding a space dimension to TFR, thereby extending it to a space-time-frequency representation (STFR). In principle, increasing the dimension of the representation is possible by considering different aspects of datasets, e.g., experimental variables; however, we herein focus on these three dimensions. These features, which are based on high temporal resolution and multichannel recording, provide detailed information on the cortical mechanisms underlying perceptions, cognition, and actions, which may be hidden in MEG and EEG data.

MEG and EEG signals themselves are recorded from sensors or electrodes, and, thus, are estimated in the source-space using various source estimation techniques with different constraints. These include an equivalent current dipole (ECD) source estimation (Sarvas, 1987; Hämäläinen et al., 1993), minimum L2 norm estimation (MNE) (Hämäläinen and Ilmoniemi, 1994), minimum current (L1 norm) estimation (MCE) (Uutela et al., 1999), and beamforming (Robinson and Varba, 1999; Gross et al., 2001; Sekihara et al., 2001). In the ECD estimation, activity is assumed to be focal, and may be explained by a small number of current dipoles. MNE is a type of distributed source modeling, which divide the source space into a grid that has a large number of dipoles (between hundreds and thousands) over the cortical surface and estimate the current in all modeled source locations at each time point with minimum power (L2-norm), resulting in data that is consistent with the measurement. MCE provides more focal estimates by using an L1-norm prior to those provided by MNE. Thus, MCE and MNE are suitable for analyzing evoked responses and examining widespread activation over time. Beamforming is also a type of distributed source modeling that acts as a spatial filter to pass brain activity from a specified location and attenuate activity from other locations. Time-domain and frequency-domain implementations of beamforming exist, e.g., a linear-constraint minimum variance beamformer (LCMV) and the dynamic imaging of coherent sources (DICS), respectively. Low-resolution electromagnetic tomography (LORETA) and its variants (e.g., eLORETA) are another family of distributed source modeling, and estimate current distributions with zero localization errors by using sparse (low spatial resolution) source configuration. Distributed source models have the advantage of no prior assumption of the source model being necessary. Recent advances in source estimation techniques have suggested a range of new algorithms as well as extended versions of early algorithms (Diwakar et al., 2011; Owen et al., 2012; Babadi et al., 2014; Huang et al., 2014; Mohseni et al., 2014). Various source estimation algorithms will be reviewed in conjunction with computations of connectivity metrics in a later section.

The brain (anatomy) has to be divided into different regions, which are used to set dipoles (or virtual sensors) for estimations of signal sources and the subsequent computation of connectivity and graph metrics. This brain parcellation may be performed on the basis on an anatomical atlas (structural parcellation) and functionally-separated regions (functional parcellation). Typical anatomical atlases include Talairach, AAL, and Harvard-Oxford atlases, whereas functional parcellation has recently been suggested for resting-state fMRI studies (Power et al., 2011; Craddock et al., 2012). Previous MEG studies used anatomical atlases to compute graph metrics (Jin et al., 2013, 2014). Another method to set dipoles within the brain is not based on any atlas. In this case, we set more dipoles with arbitrary spacing within the brain after cortical segmentation in the beamforming technique or set dipoles on cortical meshes in the minimum norm or current estimation technique. Once it has been decided how the brain is to be divided, dipoles are placed on each ROI and source estimations are then performed. Connectivity and graph metrics may also be computed from sensor-space MEG signals; however, source-space data may be more appropriate for avoiding spurious connectivity caused by a common signal source and establishing what brain regions are involved in changes in connectivity and network properties.

## Connectivity metrics

Various types of metrics may be used to quantify functional and effective connectivities for MEG/EEG signals. These metrics are used to examine relationships between two time series experimentally recorded from, for example, different brain regions, from a brain region and muscle, from cortical and subcortical regions, from a subcortical region and muscle (i.e., electromyographic activity), and from a cortical region and kinematics (i.e., acceleration of movement). These metrics also differ in linearity, the information included (amplitude and phase), volume conduction/field spread-free, and directionality (i.e., functional or effective). In the early half of this section, we provide an overview of the definition, computation, short history, and experimental comparisons of these functional connectivity metrics, and then review effective connectivity metrics.

## Functional connectivity

### Correlation

A correlation refers to a broad class of statistical relationships involving interdependence, which is any statistical relationship between two random variables or two datasets (corresponding to two time series in neuroimaging datasets). In fMRI studies, functional connectivity is an observable phenomenon that is quantifiable with measures of statistical dependencies, such as correlations and coherence (Friston, 1994). In the EEG community, correlations were frequently used to examine relationships between two EEG signals (Adey et al., 1961; Walter, 1963) even before functional and effective connectivities were introduced to fMRI data (Friston, 1994). However, at that time, the spatial blurring of electrical activity due to volume conduction and reference electrodes was already an important challenge when using correlations between two EEG time series as a metric of interregional dependence, as with coherence which is described in the next section. Electric fields generated in the brain spread in space via different conductor media (volume conduction), and, thus, EEG signals recorded by different scalp sites include an electric field derived from a common current source. Furthermore, recordings of small electric fields from the human brain require differential amplification, which needs a common, reference potential as an input. These two issues lead to a common difficulty in interpreting correlations between EEG signals. The increased strength of a common signal source obscures true interdependence between two signals recorded from two different sites. In MEG, magnetic fields are hardly affected by the conductor medium and MEG signals from different channels are not associated with any reference signal derived from a common signal source in the brain. Therefore, MEG is superior to EEG but MEG signals may still share a common cortical source (called field spread), even for planar gradiometers, despite the influence being less than in EEG. One strategy to overcome this spurious correlation is to project sensor-space data to the source space; however, signal leakage, which is an apparent signal leak between voxels in the brain space, remains due to the ill-posed nature of the inverse problem, inaccuracies in the forward solution, and incorrect assumptions caused by the inverse localization algorithm used (Brookes et al., 2014). Accordingly, correlations are generally not considered to be the best metric for examining functional connectivity in EEG and MEG, except for specific cases, e.g., distant connectivity between the cortex and another part of the body such as muscle (discussed later), the computation of correlations after specific computation (discussed later), and connectivity between the brains of different participants (hyper-scanning) (Babiloni et al., 2006; Babiloni and Astolfi, 2014; Zhdanov et al., 2015). Another important point to consider is that this issue is also inevitable to a greater or lesser extent to any non-invasive and invasive neuroimaging techniques, including fMRI, PET, NIRS, ECoG, intracerebral recordings, and LFP recordings from local regions, because signals measured by these techniques also spread in space across voxels, electrodes, and probes as in EEG and MEG.

### Coherence

Coherence is a statistic representing the relationship between two signals and is also an extension of correlation to frequency domain. Coherence (magnitude-squared coherence) is defined as:

$$\begin{array}{l} C_{xy}\left(f\right)=\frac{{\left|G_{xy}\left(f\right)\right|}^{2}}{G_{xx}\left(f\right)G_{yy}\left(f\right)} \end{array}$$

*x(t)* and *y(t)* are two time series, *G*_*xy*_*(f)* is the cross-spectral density between *x* and *y*, and *G*_*xx*_*(f)* and *G*_*yy*_*(f)* are the autospectral densities of *x* and *y*, respectively. Cross-spectra density (CSD) is calculated in the frequency domain as:

$$\begin{array}{l} C_{xy}\left(f\right)=X\left(f\right)Y^{*T}\left(f\right) \end{array}$$

Thus, CSD is the complex conjugate product of Fourier-transformed data *X(f)* and *Y(f)*. In EEG, coherence was used once (Adey et al., 1961; Walter, 1963; Sklar et al., 1972; Busk and Galbraith, 1975), but has been criticized, similar to correlations, due to issues associated with volume conduction and reference electrodes (Fein et al., 1988; Nolte et al., 2004; Nunez and Srinivasan, 2006; Stam et al., 2007b). However, coherence is applicable to some types of studies. For example, coherence has been widely and successfully used to measure functional connectivity in MEG since the introduction of the DICS method (Gross et al., 2001). Coherence is a reliable metric of functional connectivity between distant signal sources such as the cortex and muscle (Gross et al., 2001, 2005). In this case, electromyographic data recorded from peripheral muscles are used as time-series data to compute coherence. This has recently been extended to the examination of coherence between kinematics (acceleration of movement) and MEG signals (Bourguignon et al., 2013; Marty et al., 2015). Another example is demonstrated in the spino-muscle coherence of LFP in the spinal cord and electromyography in the hand muscles of conscious monkeys performing a precision motor task (Takei and Seki, 2008). A previous study investigated cortico-thalamic coherence between MEG and sub-thalamic nucleus LFP signals recorded from deep brain stimulation patients (Sander et al., 2010). It is important to note that, even for MEG, coherence may cause false positives due to the virtual zero-lag correlations caused by source leakage in functional connectivity between local regions (Brookes et al., 2014).

### Partial coherence

As described above, coherence (magnitude-squared coherence) between EEG signals is contaminated by volume conduction and reference electrodes (Rappelsberger, 1989; Andrew and Pfurtscheller, 1996; Florian et al., 1998; Mima et al., 2000). Partial coherence is computed by removing the linear effect of a common signal, as follows:

$$\begin{array}{l} {\left|R_{xy∕z}\left(f\right)\right|}^{2}=\frac{{\left|G_{xy}\left(f\right)-\frac{G_{xz}\left(f\right)G_{zy}\left(f\right)}{G\left(f\right)}\right|}^{2}}{\left(G_{xx}\left(f\right)-\frac{{\left|G_{xz}\left(f\right)\right|}^{2}}{G_{zz}\left(f\right)}\right)\left(G_{yy}\left(f\right)-\frac{{\left|G_{zy}\left(f\right)\right|}^{2}}{G_{xx}\left(f\right)}\right)} \end{array}$$

where *Gxx, Gyy*, and *Gzz* are the autospectra of EEG signals, *x, y*, and *z* for a given frequency *f* and *Gxy, Gzy*, and *Gxz* are cross-spectra between two of them. Partial coherence ranges between 0 (no association) and 1 (perfect linear association). In this computation, the signal *z* functions as a linear effect to be removed. Therefore, the signal *z* is regarded as a good reflection of a common signal that causes virtual zero-lag coherence; if not, this metric is non-sensical. Accordingly, the signal *z* must be selected from the recorded signals, such that it includes the signal derived from a common source, but not other relevant signals involved in true functional connectivity. For example, the signal from the occipital electrode may be used for *z* when examining motor-related areas in the frontal lobe. However, it is important to note that, practically, there are no ideal signals for *z* because signals from any recording sites may easily include other relevant signals that must not be removed.

### Phase coherence

An important limitation of coherence (magnitude-squared coherence) is that it only measures linear relationships between two time series and may fail to detect non-linear interdependencies between underlying dynamic systems. One possibility to overcome this issue is based on the analytical signal concept. In this approach, the instantaneous phase of time series is computed, and interactions are estimated by time-dependent *n*: *m* phase synchronization. Phase coherence is a statistical measure for phase synchronization. Synchronization was introduced to physics by Huygens in the 17th century for coupled oscillators. The concept of phase synchronization was introduced for coupled chaotic models (Rosenblum et al., 1996) and demonstrated experimentally (Parlitz et al., 1996). Phase coherence was introduced to represent the phase synchronization of EEG signals recorded from epileptic patients (Mormann et al., 2000) and is defined as:

$$R=\left|\frac{1}{N}\sum_{j=0}^{N-1}e^{i\varphi_{1,1}\left(j△t\right)}\right|=1-CV$$

where 1/Δ*t* is the sampling rate of time series and *CV* denotes the circular variance of an angular distribution obtained by transforming relative phase angles onto the unit circle in the complex plane. This metric is regarded as a specific case in which the phase locking ratio, *n*: *m*, is 1: 1, and is applicable to investigations of *n*: *m* synchronization by changing φ_1, 1_ into φ_*n, m*_. The use of Euler's formula changes the above equation into:

$$\begin{array}{l} R={\left|{\left[\frac{1}{N}\overset{N-1}{\underset{j=0}{\sum }}\sin\left[\varphi_{1,1}\left(j△t\right)\right]\right]}^{2}+{\left[\frac{1}{N}\overset{N-1}{\underset{j=0}{\sum }}\text{cos}\left[\varphi_{1,1}\left(j△t\right)\right]\right]}^{2}\right|}^{1∕2} \end{array}$$

where *R* ranges between 0 and 1. Strict phase locking results in *R* = 1, whereas a uniformed distribution of phases results in *R* = 0. A previous MEG study computed the phase coherence of two remote source activities in order to demonstrate the attentional modulation of inter-regional synchronization (Siegel et al., 2008). This study estimated source-level coherence with a two-dipole beamforming technique that allows the reliable estimation of source-level coherence, even under conditions of very high source correlation (Schoffelen et al., 2008).

### Generalized synchronization

The above approach based on the analytical signal concept is only valid if time series are approximately oscillatory, and the theory of non-linear dynamical systems provides a more general approach. The concept of generalized synchronization was introduced by Rulkov and colleagues in the context of unidirectionally coupled driver response systems (Rulkov et al., 1995). Generalized synchronization exists between two dynamical systems, X and Y, when the state of the response system, *Y* is expressed as a function of the state of the driving system, *X*, as follows:

Y=F(X)

In generalized synchronization, time series do not need to resemble each other. Several algorithms have been proposed to detect this type of interdependency in experimental time-series data; mutual false nearest neighbors (Rulkov et al., 1995), its elaborated version (Schiff et al., 1996), and Arnhold's measure (Arnhold et al., 1999). However, these metrics may be biased by the properties of individual dynamic systems (Pereda et al., 2001). Therefore, synchronization likelihood (SL) was proposed to overcome this bias (Stam and Van Dijk, 2002), and is a metric sensitive to linear and non-linear synchronization. It is also suitable for analyzing non-stationary data because it is computed in a time-dependent manner. Assuming a time series of measurements *x* and *y* (*i* = 1, …, N) recorded from *X* and *Y*, vectors are reconstructed in the state space of *X* and *Y* with time-delay embedding, as follows:

$$\begin{array}{l} X_{i}=\left(x_{i},x_{i+l},x_{i+2l},\ldots ,x_{i+\left(m-1\right)l}\right) \end{array}$$

$$\begin{array}{l} Y_{i}=\left(y_{i},y_{i+l},y_{i+2l},\ldots ,y_{i+\left(m-1\right)l}\right) \end{array}$$

where *l* is the lag and *m* is the embedding dimension. The distance between two vectors in state space is a metric of their similarity. SL *S* between *X* and *Y* is then defined at time *i*, as follows:

$$\begin{array}{l} S_{i}=\frac{1}{N}\underset{j}{\sum }\theta \left(\varepsilon_{y}-|Y_{i}-Y_{j}|\right)if\left(X_{i}-X_{j}<\varepsilon_{x}\right) \end{array}$$

where we only sum over those *j* satisfying *w*1 < |*i* − *j*| < *w*2, and *X*_*i*_ − *X*_*j*_ < ε_*x*_. *N* is the number of *j* fulfilling these conditions. *w*_1_ is the Theiler correction for autocorrelation effects and must be at least in the order of the autocorrelation time, *w*_2_ is a window that sharpens the time resolution of the synchronization metric and is set to the range of *w*_1_ ≪ *w*_2_ ≪ *N*. Thus, SL is the likelihood that the recurrence of a pattern in time series *X* at two times *i* and *j* will coincide with the recurrence of patterns in time series *Y* at the same times *i* and *j*, and takes on values between p_ref_ (no coupling) and 1 (complete coupling). P_ref_ is a parameter of the computation of SL, and is typically set to 0.01. Several variations of SL may be derived by averaging over time, space, or both. SL has been applied to EEG signals during movement observation (Calmels et al., 2010), movement execution (Calmels et al., 2008), and attention (Gootjes et al., 2006), and resting-state MEG signals for examinations of various diseases such as Alzheimer's disease (Stam et al., 2006, 2007a) or a subsequent graph-theory analysis (Stam, 2004).

### Phase locking value

Coherence may not be a pure measure of the phase relationship because it contains amplitude and phase information. The phase locking value (PLV) was introduced to detect frequency-specific transient phase locking independent of amplitude (Lachaux et al., 1999). PLV is calculated by performing a bandpass filter to extract two time series at a frequency of interest, computing its convolution (its TFR) by Wavelet or Stockwell transformation and the phase difference at each time, θ(*t*) = φ_*x*_(*t*) – φ_*y*_(*t*), and is then given by:

$$\begin{array}{l} PLV=\frac{1}{T}\overset{T}{\underset{t}{\sum }}\text{exp}\left(i\theta \left(t\right)\right) \end{array}$$

φ_*x*_(*t*) and φ_*y*_(*t*) are the phase differences of time series *x(t)* and *y(t)*. PLV calculated here reflects a single-trial phase difference, but may also be computed across trials as an average value, as follows:

$$\begin{array}{l} PLV=\frac{1}{N}\overset{N}{\underset{n}{\sum }}\text{exp}\left(j\theta \left(t,n\right)\right) \end{array}$$

where *n* indexes the *N* trials and θ (*t, n*) is the phase difference φ_1_(*t, n*) – φ_2_(*t, n*). Note that PLV is still sensitive to volume conduction/field spread and reference electrodes.

### Imaginary component of coherency

The imaginary component of coherency (iCoh) was introduced in an EEG study (Nolte et al., 2004) in order to overcome spurious zero-lag coherence. The complex coherency between two time series may be defined as the cross spectrum divided by the product of the two power spectra. Its mean over all frequencies may alternatively be computed via the mean over time of the corresponding analytical signals, as follows:

$$\begin{array}{l} c=\frac{\langle A_{1}A_{2}e^{i△\phi }\rangle }{\sqrt{\langle A_{1}^{2}\rangle \langle A_{2}^{2}\rangle }} \end{array}$$

where *A*_1_ and *A*_2_ are the amplitudes of the two time series, and Δϕ is the instantaneous phase difference between (the Hilbert transforms of) the two time series. The absolute value of coherency, typically referred to as coherence, is bounded between 0 and 1. iCoh here is given by:

$$\begin{array}{l} Im\left\{c\right\}=\frac{\langle A_{1}A_{2}sin△\varphi \rangle }{\sqrt{\langle A_{1}^{2}\rangle \langle A_{2}^{2}\rangle }} \end{array}$$

An important property of iCoh is that its (non-vanishing) finite value cannot be caused by the linear mixing of an uncorrelated source (volume conduction and reference electrode) and, thus, reflects true interactions in the source underlying the two time series. Note that iCoh depends on the amplitudes of the signals and the magnitude of the phase delay. Recent MEG studies provided evidence for the feasibility of iCoh and a relevant metric in an MEG connectivity analysis by stimulations and experiments (Sekihara et al., 2011; Ewald et al., 2012; Sekihara and Nagarajan, 2013; Cho et al., 2015; Hsiao et al., 2015).

### Phase lag index

The phase lag index (PLI) is a metric of the asymmetry of the distribution of phase differences between two signals. This metric was introduced by Stam et al. (2007b), and removes the effect of amplitude information included in iCoh. An advantage of PLI is that it is affected less by the influence of common sources due to volume conduction and/or active reference electrodes, similar to iCoh. This metric is based on the existence of a consistent, non-zero phase lag between two time series not explainable by volume conduction from a single source, and, as a consequence, renders true interactions between the underlying systems likely. Therefore, PLI may be considered to be the same to PLV as iCoh is to coherence (Brookes et al., 2014). Such consistent, non-zero phase lags may be determined from the asymmetry of the distribution of instantaneous phase differences between two signals.

$$\begin{array}{l} PLI=|\langle sign\left[△\phi \left(t_{k}\right)\right]\rangle | \end{array}$$

PLI ranges between 0 and 1. A PLI of zero indicates either no coupling or coupling with a phase difference centered around 0 and π. A PLI of 1 indicates perfect phase locking at a value of Δϕ, different from 0 mod π. The stronger this non-zero phase locking is, the larger PLI will be. PLI has no information about the leading phase. However, this information is easily recovered by disregarding the absolute value in the equation above. Surrogate data may be introduced to determine whether PLI is significantly larger than zero. In brief, PLI has to be computed for both the original time series of a set of surrogate data that match the original data, but lacks any correlations between channels, e.g., by shifting each channel by some random phase. Differences between the PLI of original and surrogate data yield z-scores that are sufficient for defining significance levels. It is important to note that the equation of PLI requires the phase difference to be bounded in the interval −π < Δϕ < π. In contrast, if phases are defined in the interval 0 < Δϕ ≤ 2π, then the equation needs to be modified to:

$$\begin{array}{l} PLI=|\langle sign\left[sin△\phi \left(t_{k}\right)\right]\rangle | \end{array}$$

### weighted PLI (wPLI) and debiased wPLI (dwPLI)

The PLI of sensitivity to noise and volume conduction may be hindered by discontinuity because perturbations turn phase lags into leads and vice versa. This has more serious implications for the synchronization effects of a small magnitude. Therefore, weighted PLI was developed in order to increase the capacity to detect true changes in phase synchronization, reduce the influence of common noise sources, and reduce the influence of changes in the phase of coherency (Vinck et al., 2011). In weighted PLI, the contribution of the observed phase leads and lags is weighted by the magnitude of the imaginary component of the cross-spectrum.

$$\begin{array}{l} \Phi \equiv \frac{\left|\left\{ℑ\left\{X\right\}\right\}\right|}{E\left\{\left|ℑ\left\{X\right\}\right|\right\}}=\frac{\left|E\left\{|ℑ\left\{X\right\}|sgn\left(ℑ\left\{X\right\}\right)\right\}\right|}{E\left\{\left|ℑ\left\{X\right\}\right|\right\}} \end{array}$$

The numerators of wPLI and magnitude of iCoh are the same, with the only difference being normalization in the denominator. Hence, wPLI has 2 advantages over PLI; one is a reduction in sensitivity to additional, uncorrelated noise sources, while the other is an increase in the statistical power to detect changes in phase synchronization. Furthermore, the debiased version of wPLI (dwPLI) has been suggested to reduce sample-size bias, which is another factor affecting phase synchronization. dwPLI is defined as:

$${\hat{\Omega }}^{W}\equiv \frac{\sum_{j=1}^{N}\sum_{k\neq j}ℑ\left\{X_{j}\right\}ℑ\left\{X_{k}\right\}}{\sum_{j=1}^{N}\sum_{k\neq j}\left|ℑ\left\{X_{j}\right\}ℑ\left\{X_{k}\right\}\right|}$$

This estimator may also be written as a weighted statistic:

$${\hat{\Omega }}^{W}=\frac{\sum_{j=1}^{N}\sum_{k\neq j}W_{j,k}d\left(X_{j},X_{k}\right)}{N\left(N-1\right)\bar{W}}$$

where the weight $W_{j,k}\equiv \left|ℑ\left\{X_{j}ℑ\left\{X_{k}\right\}\right\}\right|$, $\bar{W}$ is the average weight, called weight normalization, defined as $\bar{W}\equiv \frac{1}{N(N-1)}\sum_{j=1}^{N}\sum_{k=j+1}W_{j,k}$, and the function *d* is defined as:

$$\begin{array}{l} d\left(U,V\right)\equiv sgn\left(ℑ\left\{U\right\}\right)sgn\left(ℑ\left\{V\right\}\right) \end{array}$$

where *U* and *V* are complex-valued random variables. Normalized weight is defined by the expression $W_{j,k}∕\left(N\left(N-1\right)\bar{W}\right)$ and sums up to one. Thus, dwPLI is obtained by computing the imaginary component of the cross-spectra, computing the average imaginary component of the cross-spectra, and normalizing by the computed average over the magnitudes of the imaginary components of cross-spectra. If all weights *W*_*j, k*_ equal 1, then this equals the unbiased PLI-square estimator. dwPLI, wPLI, PLI, and other metrics were compared in LFP data recorded from the orbitofrontal cortex of the monkey (Van Wingerden et al., 2010).

### Band limited power correlation

A band limited power or amplitude envelop correlation provides functional coupling without coherence or phase coherence (Bruns et al., 2000). One typical approach for computing band limited amplitude or power envelope uses a Hilbert transform. Once the time series for each voxel in the source space is obtained by a source estimation of recorded magnetic fields, its analytic signal is then expressed using the Hilbert transform of the time series. The envelope of oscillatory activity is obtained by measuring the magnitude of the analytic signal, i.e., Hilbert envelopes. Pearson's correlation between Hilbert envelopes at different voxels is then computed as a metric of functional connectivity. This kind of correlation has been applied to ECoG signals (Bruns et al., 2000; Nir et al., 2008), MEG signals from normal and blind subjects (Brookes et al., 2011; Schepers et al., 2012), and LFP signals in monkeys (Leopold et al., 2003) and humans (Nir et al., 2008). Several studies have employed ICA to determine what signals need to be used in order to compute the connectivity of band-limited power time series. ICA itself is a linear family of blind source separation methods; however, it is often applied in MEG studies to non-linear, band-limited power time series (Brookes et al., 2011; Luckhoo et al., 2012; Hall et al., 2013). Accordingly, this may also be regarded as a non-linear approach (Brookes et al., 2014). It has also been reported that changes in oscillatory power in MEG/EEG are associated with fMRI BOLD responses (Scheeringa et al., 2011). A recent study demonstrated that an fMRI BOLD correlation reflected a frequency-specific neuronal correlation of source-space signals in MEG data (Hipp and Siegel, 2015). Accordingly, the examination of power correlations is important when considering the relationship between the neuronal mechanisms underlying recorded MEG signals and those of BOLD signals.

### Power envelope correlation of orthogonalized signals

Signal components that reflect the same source at two different sensors (or source estimates) are characterized by an identical phase. In contrast, for many cases, signals from different neuronal populations may be considered to have a variable phase relationship. This difference is used to remove the spurious zero-lag correlation of MEG signals caused by field spread in the sensor-space and signal leakage in the source-space (Hipp et al., 2012). In order to compute this, the orthogonalization of raw signals, i.e., removing the linear regression, is initially performed, which corresponds to the removal of signal components sharing the same phase, for each pair of signals, time window and carrier frequency. After orthogonalization, power envelopes are computed, and the linear correlation between these power envelopes is then computed as a measure of connectivity. This computation ensures that the signals do not share a trivial correlation in power resulting from methodological issues. Furthermore, a relevant technique, a residual envelope correlation, which has been extended from residual coherence, was recently suggested (Sekihara and Nagarajan, 2013).

### Mutual information

Mutual information (MI) is an information-theoretic metric of the mutual dependence of two random variables, and may be regarded as a decrease in uncertainty regarding one variable when knowledge of another is given. MI is based on Shannon entropy, which is a measure quantifying the uncertainty of a probability distribution. Shannon entropies of the marginal distributions of the systems *X* and *Y*, and of the joint distribution are defined as:

$$\begin{array}{l} H\left(X\right)=-\overset{M_{x}}{\underset{i=1}{\sum }}p_{x\left(i\right)}{\text{log}p}_{x}\left(i\right) \end{array}$$

$$\begin{array}{l} H\left(Y\right)=-\overset{M_{y}}{\underset{i=1}{\sum }}p_{y\left(i\right)}{\text{log}p}_{y}\left(i\right) \end{array}$$

$$\begin{array}{l} H\left(X,\;Y\right)=-\overset{M_{x}}{\underset{i=1}{\sum }}\overset{M_{y}}{\underset{j=1}{\sum }}p_{xy\left(i,\;j\right)}{\text{log}p}_{xy}\left(i,j\right) \end{array}$$

MI is then defined as:

$$\begin{array}{l} I\left(X,Y\right)=H\left(X\right)+H\left(Y\right)-H\left(X,Y\right) \end{array}$$

MI is also interpreted as Kullback-Leibler entropy by measuring the gain the information when replacing the distribution of *p*_*x*_(*i*)*p*_*y*_(*j*) by the actual joint distribution *p*_*xy*_(*i, j*), as follows:

$$\begin{array}{l} I\left(X,\;Y\right)=-\overset{M_{x}}{\underset{i=1}{\sum }}\overset{M_{y}}{\underset{j=1}{\sum }}p_{xy\left(i,\;j\right)}\text{log}\frac{p_{xy}\left(i,j\right)}{p_{x}\left(i\right)p_{y}\left(j\right)} \end{array}$$

The base of the logarithm determines the units in which information is measured. A common example is to take base two, which means information to be measured in bits. The classical and common strategy to estimate MI from two time series, *x* and *y* involves partitioning the supports of *X* and *Y* into bins of finite size and counting the numbers of points falling into the various bins. MI has been successfully applied to MEG signals for speech processing (Cogan and Poeppel, 2011), cortical processing in the blind (Ioannides et al., 2013), or those with developmental disorders (Gómez et al., 2014), a graph-theory analysis of resting-state data (Jin et al., 2011, 2014), and musical expertise (Paraskevopoulos et al., 2015), as well as to EEG signals. In most of the above studies, the classical strategy was used to estimate MI, while other techniques (Kraskov et al., 2004) and correction techniques are available to overcome the binning bias issue (Roulston, 1999; Zhu et al., 2014).

### Comparisons of functional connectivity metrics

The metrics of functional connectivity described above differ from each other in sensitivity to a bias by common signal sources, linearity or non-linearity, and the included phase and amplitude information. iCoh, the PLI family, and power envelope correlation are less sensitive to spurious zero-lag connectivity caused by common cortical sources, including volume conduction, field spread, reference electrodes, and source leakage, and, thus, reflect true functional connectivity. Correlations, coherence, phase coherence, and PLV may provide spurious zero-lag connectivity between different cortical regions. However, there are several conditions to which the latter metrics are applicable for examining functional connectivity; e.g., cortico-muscular connectivity (Gross et al., 2001, 2005), cortico-kinematic connectivity (Bourguignon et al., 2013; Marty et al., 2015), and spino-muscle connectivity (Takei and Seki, 2008). However, iCoh, the PLI family, and power envelope correlation may miss linear, but functionally meaningful interactions, which, in principle, may be expressed in zero or near-zero lag coherence. In spite of this potential omission, these metrics are free of any artifact of volume conduction, and do not cause type 1 errors (false positives). In contrast, other metrics such as correlations, coherence, and PLV may cause type 1 errors.

## Effective connectivity

The functional connectivity metrics described above provide no directed flow of information or causality, whereas various methods have been suggested to infer directed information. We briefly summarize these below.

### Granger causality index

Granger causality is a popular statistical concept of causality, which has been applied to several types of neuroscientific data in humans, non-human primates, and rodents. Interactions between two stochastic processes (or two signals) *X* and *Y* with the discrete time series *x*(*t*) and *y*(*t*) may be described by bivariate linear autoregressive (ARX) models:

$$\begin{array}{l} x\left(t\right)=\overset{q}{\underset{k=1}{\sum }}a_{11,k}x\left(t-k\right)+\overset{p}{\underset{k=1}{\sum }}a_{12,k}y\left(t-k\right)+e_{x}\left(t\right) \end{array}$$

$$\begin{array}{l} y\left(t\right)=\overset{q}{\underset{k=1}{\sum }}a_{21,k}x\left(t-k\right)+\overset{p}{\underset{k=1}{\sum }}a_{22,k}y\left(t-k\right)+e_{y}\left(t\right) \end{array}$$

where *p* and *q* are the model orders of *y* and *x* regressors, *e*_*x*_(*t*) and *e*_*y*_(*t*) are the model prediction errors (residuals), and each is assumed to be serially uncorrelated over time. If the variance of *e*_*x*_(*t*) is reduced by adding the *Y* terms in the first equation, *Y* Granger-causes *X*, and vice versa. In other words, *Y* Granger-causes *X* if the coefficients in *a*_12_ are jointly significantly different from zero. This may be examined by performing an *F*-test of the null hypothesis that *a*_12_ = 0, given assumptions of covariance stationarity on *X* and *Y*. The degree of a Granger causality interaction is estimated by the logarithm of the corresponding F-statistic. The Akaike Information Criterion (AIC) or Bayesian Information Criterion (BIC) is used to determine the appropriate model order.

### Dynamic causal modeling

Dynamic causal modeling (DCM) was initially introduced in an fMRI study (Friston et al., 2003). DCM is a metric of causality, and enables us to examine how brain areas within a network interact. It is a generic Bayesian framework for inferring hidden neuronal states from measurements of hemodynamic or electromagnetic activity in the brain. DCM models the cognitive system at its underlying neuronal level (called the neuronal mass model). The modeled neuronal dynamics are transformed into area-specific BOLD signals by a hemodynamic model. The aim of DCM is to estimate parameters at the neuronal level such that the modeled and measured BOLD signals are optimally similar. The first introduction of DCM was a bilinear version (Friston et al., 2003), and a non-linear version is also now available (Stephan et al., 2008). DCM is also available for MEG/EEG signals (FitzGerald et al., 2015; Garrido et al., 2015; Klingner et al., 2015). Studies utilizing DCM have investigated a wide range of topics; motor network (Volz et al., 2015), action observation (Thioux and Keysers, 2015), sense of agency (Ritterband-Rosenbaum et al., 2014), attention (Brown and Friston, 2013), handedness (Pool et al., 2014), cognitive control and working memory (Harding et al., 2015), naturalistic communication (Regenbogen et al., 2013), neurodevelopmental disorders (Albouy et al., 2015), steady-state visual evoked responses with simultaneous EEG-fMRI recordings (Youssofzadeh et al., 2015), and applications to ECoG signals in the monkey (Bastos et al., 2015).

### Causality metrics in the frequency domain

As discussed above, although Granger causality is a statistic of causality computed in the time domain, relevant statistics in the frequency domain may also be calculated, including directed transfer function (DTF) (Kaminski and Blinowska, 1991), partial directed coherence (PDC) (Baccalá and Sameshima, 2001), and spectral Granger causality (Geweke, 1982) metrics. A non-linear PDC was recently suggested and applied to EEG signals from an epileptic patient (He et al., 2014).

### Transfer entropy

Transfer entropy is an information-theoretic measure of time-directed information transfer between two random processes (Schreiber, 2000). TE measures the rate of information flow between two variables as a violation of the Markov property, which is a memoryless property of a stochastic process. TE from two time series *x*_*t*_ and *y*_*t*_ is written as:

$$\begin{array}{l} \text{TE}\left(X\to Y\right)\\ =\underset{y_{t\;+\;u},{\text{y}}_{t}^{d_{y}},{\text{x}}_{t}^{d_{x}}}{\sum }p\left(y_{t+u},{\text{y}}_{t}^{d_{y}},{\text{x}}_{t}^{d_{x}}\right)log\frac{p\left(\left(y_{t+u}|{\text{y}}_{t}^{dy}{\text{x}}_{t}^{dx}\right)\right)}{p\left(y_{t+u}|{\text{y}}_{t}^{d_{y}}\right)} \end{array}$$

where *t* is a time index, *u* is the prediction time, and ${\text{y}}_{t}^{d_{y}}$ and ${\text{x}}_{t}^{d_{x}}$ are *d*_*x*_- and *d*_*y*_-dimensional delay vectors. In contrast to Granger causality relating to prediction, TE is framed in terms of the resolution of uncertainty. The Granger Causality family described above is also a data-driven, model-based metric of causality and DCM is a biologically inspired, model-based approach, whereas TE is a model -free method. Thus, TE is advantageous for exploratory analyses over the Granger causality and other model-based methods, especially for the detection of unknown non-linear interactions. TE fulfills several important requirements for detecting causal relationships between two time series in neuroscience (Vicente et al., 2011). It does not require the *a priori* definition of the type of interaction. It may detect frequently observed types of purely non-linear interactions. Furthermore, TE detects causal relationships even when a wide distribution of delays exists in interactions between two time series. TE is also robust against linear cross-talk between time series. TE has been applied to MEG and EEG studies on auditory speech (Park et al., 2015), simple motor tasks (Vicente et al., 2011), auditory working memory tasks (Wibral et al., 2011), and an epileptic patient (Chávez et al., 2003). Early fMRI, EEG, and MEG studies (Hinrichs et al., 2006, 2008) used a very similar measure, called directed information transfer (DIT), on the basis of directed transinformation (Saito and Harashima, 1981; Kamitake et al., 1984); however, this computation appears to limit the detection of directed interactions to linear ones (Vicente et al., 2011). In addition, a multivariate extension of transfer entropy and MI (Lizier et al., 2011) and a phase version of TE have been proposed (Lobier et al., 2014).

### Phase slope index

The phase slope index (PSI) is a metric used to estimate the direction of information flux during multivariate time series from complex systems and was proposed by Nolte and colleagues (Nolte et al., 2008b). This metric is based on the slope of the phase spectrum, and has invariance properties that are important for applications in real physical or biological systems. PSI is strictly insensitive to mixtures of arbitrary independent sources, gives meaningful results even if the phase spectrum is not linear, and properly weighs contributions from different frequencies. Compared with standard Granger causality, PSI has the ability to detect true interactions, even in mixed and noisy complex systems. In the Causality Challenge, PSI provided a higher score (+578) than Granger causality (−264) (Nolte et al., 2008a). It has also been applied to source-space EEG data (Nolte and Müller, 2010).

## Appropriate metrics of connectivity

The question of what metric is preferable for representing inter-regional relationships is ultimately a statistical issue, and also depends on the research of interest. This choice will depend on which types of errors, false positives and negatives, are acceptable for the respective research and whether researchers take the risk of making either error. Another point of view is associated with the specific research question about interdependence between signals, regions, or voxels, namely, what type of interdependence between two signals are researchers interested in: correlation, phase, or directed influence. This means that researchers need a clear scientific hypothesis and the resulting experimental hypotheses that determine what type of metrics should be used. It is important to consider these differences including advantages and disadvantages and to employ appropriate measures for individual research. An opposite point of view on the selection of metrics to be used is also important. For example, researchers must choose an arbitrary one of connectivity metrics on the basis of ambiguous information in an exploratory study that makes it difficult to determine the metric to be used prior to the experiment. Although this is an inefficient approach, it does exist. This is important for effective connectivity metrics because some of these need the *a priori* definition of the type of interaction to be computed; however, researchers often cannot obtain *a priori* information, especially in a large-scale data set. TE or other variants may be more appropriate in such exploratory cases of effective connectivity.

## Various source estimation algorithms used in connectivity studies

Various source estimation algorithms are currently available, as described partly in the Basics of MEG/EEG section; however, computations of connectivity from MEG/EEG data require time-series data or converted frequency-series data. Accordingly, source estimation algorithms to preprocess the computation of source-space connectivity may fulfill some requirements; e.g., (1) the reconstruction of time-series data in the source space is possible, (2) the number of estimated sources in addition to the location and orientation of each source are accurate, and (3) computation demand is as low as possible. Some algorithms themselves provide source-space connectivity, e.g., the DICS algorithm reported by Gross and colleagues regarding coherence, as described in the earlier Coherence section. According to Gross et al. (2010), at least three strategies are available for identifying significant connectivity pairs in the source space (Gross et al., 2010); (1) the selection of individual anatomical brain regions based on a priori information such as findings from other studies and subsequent tests for the connectivity of time-series data between selected regions, (2) the selection of regions based on their activity and subsequent tests for connectivity, and (3) the selection of regions directly based on their connectivity. Most of the available source reconstruction algorithms belong to the second category, whereas DICS belongs to the third category. The opposite strategy for connectivity studies is the brain-wide computation of connectivity in the source space, i.e., computations of connectivity for all pairs of available voxels (or meshes, virtual sensors, and dipoles).

As distributed source estimation algorithms, there are minimum-norm techniques, magnetic field tomography (MFT), and beamforming available for subsequent computations of connectivity. Regarding minimum-norm techniques, one previous study filtered MEG signals with a Morlet wavelet and then reconstructed sources on a single-trial basis with dynamic statistical parametric mapping (dSPM) (Lin et al., 2004). This study showed power estimates normalized by the baseline value on a cortical surface without selecting regions of interest, and PLV was also mapped as an index of phase synchronization. David and colleagues detected active voxels using a similar source reconstruction algorithm, but with surrogate data. Non-averaged data were used for source localization, and the iteration procedure employed reduced the number of active voxels, which provided the sparse and focal source representations used for computations of connectivity (David et al., 2002, 2003). Minimum-norm source reconstruction was also applied to subsequent computations of the frequency flow metric, which is based on an estimation of the instantaneous frequency in the time-frequency plane (Amorim et al., 2005; Rudrauf et al., 2006). In another study, the DTF (Kaminski and Blinowska, 1991) was also computed as an index of effective connectivity after MNE (Astolfi et al., 2005). Synchronization tomography is another class consisting of a source estimation algorithm and subsequent computation of phase synchronization (Tass et al., 2003). Source estimation is based on MFT, a non-linear localization algorithm, to compute current densities with the brain for each recorded data sample (Ioannides et al., 1990; Poghosyan and Ioannides, 2008). MFT may be applied to the non-averaged data used to compute subsequent MI and time-delayed MI as an index of functional connectivity (Ioannides et al., 2000, 2004, 2013).

Other algorithms are based on the ECD estimation with relatively small numbers of ECDs. These consist of computations of source coherence using a multidipole model (Hoechstetter et al., 2004) and phase synchronization of independent components (ICs) separated from recorded data using an independent component analysis (ICA) implemented in EEGLAB (Makeig et al., 2004a,b). A recent source estimation algorithm, called *Champagne*, uses a novel empirical Bayesian scheme to estimate the number and location of a small (sparse) set of flexible dipoles that adequately account for the observed sensor-space data (Owen et al., 2009, 2012; Wipf et al., 2010). These studies compared the abilities of *Champagne*, MCE, beamforming, and sLORETA/dSPM to detect connectivity with coherence, iCoh, and multivariate autoregression (MVAR). MVAR is a valuable tool because it quantifies the time scale of interactions and provides directionality by modeling data as the linear mixing of current and past values of the data. An empirical analysis with various datasets showed that *Champagne* outperformed other estimation algorithms in terms of localization accuracy and time-course reconstruction at various signal-to-noise ratios. Therefore, the high suitability of this algorithm for functional connectivity analyses was suggested because it is robust to highly correlated dipoles and circumvents the issue of computational complexity by vastly pruning the number of active voxels (only 4–7 dipoles were estimated and used to compute connectivity metrics). Consequently, *Champagne* also functions to reduce the spatial dimension of data on the basis of the MEG signal itself, which is reasonable for computing functional connectivity in terms of decreasing computational demand and avoiding source leakage. Thus, sparse source configurations including *Champagne* are very useful for examining connectivity. In contrast, connectivity may need to be computed for all pairs of as many dipoles as possible (more than one thousand) in order to prevent any sign of small interactions being missed, and also to examine graph metrics computed from the brain-wide connectivity matrix. Both approaches are highly valuable from a scientific point of view, and, thus, differentiating between them is also considered important. *Champagne* is well suitable for source-space functional connectivity under various experimental conditions, in which only a small number of activated regions are predicted, and may be applied to an examination of instantaneous connectivity. In contrast, brain-wide computations of source-space connectivity and a subsequent network analysis requires source reconstructions by beamforming or MCE, MNE naturally, and the use of non-instantaneous connectivity metrics in order to avoid spurious connectivity between local regions.

## EEG-TMS technique for effective connectivity

Another technique to examine effective connectivity involves the integrative use of recordings of brain activity and stimulation of the brain region. A typical example is the EEG-TMS technique (Ilmoniemi et al., 1997). By recording EEG responses to a transcranial stimulation of the brain region, the EEG-TMS technique allows us to estimate the time course of the causal effects of the stimulated regions on other regions in which the recorded responses may originate. This technique has been applied to studies on attention (Morishima et al., 2009), task and decision inertia (Akaishi et al., 2010, 2014), and perceptual decision making (Akaishi et al., 2013). Similar techniques are also possible in other recording modalities, such as PET-TMS (Paus et al., 1997), fMRI-TMS (Bestmann et al., 2004), and NIRS-TMS (Noguchi et al., 2003). These techniques are based on artificial neuronal impulses evoked by TMS, but are also applicable to examinations of the actual involvement of the stimulated and recorded regions by integrating with the virtual lesion technique. In the near future, these techniques may be integrated with connectivity metrics, which were reviewed above, and/or graph metrics, which are reviewed below. This integration may open a new avenue for examining the dynamics of activity magnitude, connectivity, network, and the actual involvement of brain regions. The invasive type of this technique is also available (Matsumoto et al., 2012). A comprehensive consensus paper (Siebner et al., 2009) and Research Topic in Frontiers (“Manipulative approaches to human brain dynamics” edited by Kitajo, Hanakawa, Ilmoniemi, and Miniussi) are helpful for learning more about these integrative techniques.

## Network or graph metrics derived from a graph-theoretical analysis

In previous sections, we discussed various functional and effective connectivity metrics computed from functional brain signals, which basically describe various statistical relationships between two signals and are a collection or averaged representation of only a very small fraction of a network. The third category (network or graph) provides higher-level perspectives of functional organization in the human brain network. A graph-theoretical analysis computes metrics describing the various local and global properties of a network, and takes many connectivity values into consideration. This powerful analysis of neuroimaging data has revealed interesting network properties including small-world organization, scale-free features, hubs, modularity, and rich clubs. A typical method used to obtain graph metrics is to initially compute connectivity values between two signals for all pairs of nodes, and then perform a graph-theoretical analysis on the connectivity matrix obtained. In an unweighted graph, connectivity values are thresholded with certain criteria and binarized before the computation of graph metrics, while weighted graph metrics are computed from an original connectivity matrix without thresholding. This analysis has been applied to structural and functional MRI data, and detected the structural core in the posterior medial and parietal regions, which markedly overlapped with the default-mode network (Hagmann et al., 2008). Its application to macaque data has also been reported (Honey and Sporns, 2008). This map of neural connections within the brain is now regarded as a connectome (Hagmann, 2005; Sporns et al., 2005). The first application of the graph theory to fMRI data demonstrated various clusters in the form of subgraphs in a finger tapping task (Dodel et al., 2002). Stam (2004) first performed a graph-theoretical analysis on resting-state MEG datasets in the sensor-space (Stam, 2004), and subsequently on resting-state EEG datasets (Stam et al., 2007a). Bassett and colleagues also reported a small-world architecture together with the fractal organization using sensor-space MEG data (Bassett et al., 2006). This has provided an abundant amount of evidence to support alterations in network properties in various neurological diseases (Stam, 2014). We herein briefly summarize relevant important graph metrics. Please refer to recent review articles for more details (Bullmore and Sporns, 2009; Rubinov and Sporns, 2010; Sporns, 2013; Sporns and Bullmore, 2014; Stam, 2014).

### Degree

The degree of the vertex of a graph is the number of edges incident to the vertex, with loops counted twice. Thus, individual values of the degree characterize the importance of vertices in the network. The degree distribution is the probability distribution of these degrees over the whole network, and an important metric of network development and resilience. The mean network degree is commonly used as a metric of density, or the total wiring cost of the network (Rubinov and Sporns, 2010). In a directed graph, nodes have two different degrees, the in-degree and out-degree, which are the numbers of incoming and outgoing edges, respectively. In a weighted graph, instead of the degree, the same type of metric is called the strength, which equals the sum of all neighboring edge weights.

### Clustering coefficient and transitivity

A clustering coefficient is a metric of the degree to which vertices in a graph cluster together. There are two modes of clustering coefficients; local and global. The local clustering coefficient of a vertex is the proportion of edges between vertices within its neighborhood divided by the number of edges that may exist between them (Watts and Strogatz, 1998). Accordingly, the local clustering coefficient is 1 if the neighborhood is fully connected, whereas the value is 0 if there are no connections in the neighborhood. Therefore, this value represents how well connected the neighborhood of the node is (“cliquishness”). The average of local clustering coefficients across all vertices in a graph, often called the mean clustering coefficient, reflects the overall level of clustering in a network (Watts and Strogatz, 1998). This means the clustering coefficient is also regarded as an alternative to the global clustering coefficient (also called transitivity), which is a classical metric to measure the overall level of clustering (Luce and Perry, 1949; Wasserman and Faust, 1994). The local clustering coefficient is one of the factors determining the small-world feature of a network by Watts and Strogatz, while another is the characteristic path length. The ratio of the local clustering coefficient and weighted averaged shortest path length is called small-worldness, and has been applied to MEG signals (Stam et al., 2009; Douw et al., 2011).

### Characteristic path length

The characteristic path length is the average shortest path length between all pairs of nodes in a network (Watts and Strogatz, 1998). As the name suggests, the shortest path is defined as the shortest path from one vertex to another in a network, which is computed as the least number of or least total weight of edges. Various algorithms have been suggested to compute the shortest path, including Dijkstra's algorithm, its modifications, the Bellman-Ford algorithm, Gabow's algorithm, the Floyd-Warshall algorithm, Pettie-Ramachandran algorithm, and Johnson algorithm.

### Local efficiency

The efficiency of a network is a metric of the efficiency of information exchange in network science. Local and global types of efficiency have been identified. Local efficiency is computed as the inverse of the shortest path length between connected vertices that are neighbors with the vertex of interest (Latora and Marchiori, 2001). Therefore, local efficiency quantifies how well information is exchanged by its neighbors when it is removed, and, thus, is a metric of the resistance of a network to failure at the local level. This is also regarded as an alternative to the local clustering coefficient.

### Global efficiency

Global efficiency is computed by dividing the average efficiency of a graph with that of an ideal graph, which is comparable to the inverse of the average path length of a network (Latora and Marchiori, 2001). However, the inverse of the average path length measures efficiency in a system in which one packet information is being moved via the network, whereas global efficiency measures efficiency when all the vertices are exchanging packets of information with each other. Global efficiency was previously shown to be easier to use numerically than the path length (Bullmore and Sporns, 2009). Local and global efficiency metrics are valuable when examining physical biological networks.

### Modularity

Modularity is a metric that describes the structure of a network, and is computed as the fraction of the edges that fall within the given groups minus the expected fraction if edges were randomly distributed. Thus, modularity represents the degree to which a network is subdivided into non-overlapping groups (also called modules, clusters, and communities). Computation techniques for modularity utilize optimization algorithms (Newman, 2006; Blondel et al., 2008; Leicht and Newman, 2008). Other algorithms also detect overlapping community structures (Palla et al., 2005).

### Degree centrality

Important brain regions, sometimes called hubs or cores, interact with other regions, and may be the center of information flow. This may be expressed by how many individual vertices connect with others or how many of the shortest paths the vertex is involved in. Degree centrality is one of the most common metrics of centrality. Vertices with a high degree of centrality connect with more vertices than those with a low degree of centrality.

### Closeness centrality and betweenness centrality

Closeness centrality represents how close individual vertices are to others on average in a network, and is computed as the inverse of the average shortest path length from a vertex to all other vertices in the network (Sabidussi, 1966; Freeman, 1978). Betweenness centrality is a metric of the importance of the vertex in a network (Freeman, 1978). This metric is a computationally expense among centrality metrics but is very popular. A large number of neuroimaging studies have successfully used betweenness centrality to reflect the importance of a network (Hipp et al., 2012; Kida and Kakigi, 2013; Kida et al., 2013) and degree distribution. Computations of betweenness centrality and closeness centrality are both based on the computation of short paths. Several algorithms including Brandes's (Brandes, 2001) and Kintali's algorithms (Kintali, 2008) have been proposed in order to compute betweenness centrality. Closeness and betweenness centralities are based on central vertices being involved in many of the shortest paths in a network, and, consequently, function as important controls of information flow (Freeman, 1978; Rubinov and Sporns, 2010).

### Motifs

A motif is a generalized version of the clustering coefficient, and a pattern of local connectivity. The presence of a motif is demonstrated by showing the frequent occurrence of that pattern in the network, and is actually examined with the motif z-score compared with the surrogate data (Milo et al., 2002).

### Small-worldness

The clustering coefficient and averaged shortest path length are metrics representing the small-world feature of a network. A second-order graph statistic, which is a metric of small-worldness, is computed by dividing the ratio of the clustering coefficient in relation to surrogate data by that of the averaged shortest path length in relation to surrogate data (Humphries and Gurney, 2008). This metric has been used in sensor-space MEG data (Douw et al., 2011).

## Brain-wide analysis of connectivity and graph metrics

In recent years, MEG studies have conducted a brain-wide source-space analysis of resting-state functional connectivity (De Pasquale et al., 2010; Brookes et al., 2011; Hipp et al., 2012) and graph metrics (Hipp et al., 2012). Since connectivity is as transient, adaptive, and context-sensitive as brain activity *per se* (Friston, 2011), another important issue in MEG/EEG studies is rapid changes in graph metrics during task states in the source-space. Bassett and colleagues reported task-related changes in the degree and betweenness centrality of the gamma-band network in the sensor space with the execution of visually-initiated continuous finger tapping at 1.2 Hz for approximately 10 s (4 repetitions for each participant) compared to a resting condition. This does not measure rapid changes in graph metrics, while recent studies reported brain-wide, task-related temporal changes in graph metrics computed from full sensor-space data in a multisensory attention task (Kida and Kakigi, 2013) and working memory task (Ariza et al., 2015). A recent EEG study reported brain-wide network properties computed from source-space data in a visual discrimination task (Bola and Sabel, 2015). However, most EEG/MEG studies performing the computation of graph metrics in the source-space have divided the brain into approximately 100 or fewer regions (voxels or grid points, meshes) based on various brain atlases (such as AAL, Talairach, and Harvard-Oxford) in order to compute source activity, connectivity, and graph metrics. In contrast, the first demonstration of the human structural connectome using a graph-theory analysis by Hagmann and colleagues was provided based on a computation with 998 vertices (Hagmann et al., 2008). The brain-wide graph-theory analysis of resting-state MEG by Hipp and colleagues was performed with 2926 vertices in the source space (Hipp et al., 2012). Therefore, larger numbers of vertices are preferable for computing the brain-wide analysis of task-related, rapid changes in graph metrics in MEG and EEG studies. A preliminary MEG study reported task-related, frequency-specific, rapid temporal changes in the brain-wide network organization by examining graph metrics computed from full source-space data (about 900 vertices) in a multisensory attention task (Kida et al., 2013). The findings of this study indicated the possibility of the cortical dynamics of functional networks showing frequency-specific, sub-second rapid temporal changes depending on the periods of task execution. fMRI studies have also been investigating brain-wide task-related temporal changes in graph metrics (Wang et al., 2013). This type of exhaustive analysis handling a full data space, which includes several categories of neural indices in space, time, and frequency dimensions, is an extremely time-consuming task with personal computers or workstations and may feasibly be achieved using parallel computing or GPU computing, besides supercomputers. In contrast, dimensional reductions in source space data such as a minimum spanning tree or clustering makes the computations of graph metrics efficient. Thus, advances in this line of research will undoubtedly lead to more exciting discoveries on brain mechanisms based on the activity strength, connectivity, and network along different dimensions (space, time, and frequency).

## Complex dynamics of the human brain

Brain signals with high-temporal resolution such as MEG are feasible for examining dynamical systems in the brain, which works rapidly and adaptively. Many events in the real world show complex behaviors that cannot simply be explained by linear equations, whereas others can. Complex systems is a collective term to describe a system showing various properties and functions such as fractal (self-similarity), chaos, cellular automaton, percolation, and self-organized criticality, by large-scale interactions or strong non-linearity between elements involved in the system. Complex systems have a wide variety of real-world examples including social organization, artificial intelligence, ecosystems, earthquakes, living cells, the universe and, of course, the brain. The identification of complex systems in the human brain has been associated with connectivity and network analyses, and, thus, we briefly review MEG/EEG studies characterizing complex dynamics in the human brain.

### Fractal scaling

Fractals are a measure of self-similarity, which is an important concept characterizing complex dynamics. fMRI studies performed the fractal scaling of low frequency human brain activation, mainly in a resting state (Bullmore et al., 2004; Maxim et al., 2005; Achard et al., 2008; Suckling et al., 2008; Wink et al., 2008). Classical studies reported the feasibility of a fractal analysis of stereo-EEG (SEEG) signals directly recorded from the cortex of epileptic patients for a diagnosis (Bullmore et al., 1994). Bassett and colleagues used MEG to demonstrate that human brain oscillations showed scale-invariant or fractal scaling over the frequency range of 1–75 Hz (Bassett et al., 2006). MEG signals recorded using a 275-ch whole head system (axial type) in a resting state and during finger tapping, and computed graph metrics (average path length, clustering, sigma, characteristic path length), synchronizability, and dynamic parameters (such as the power law exponent and exponential cut-off degree). All these metrics showed scale invariance or fractal scaling over the frequency range. However, major changes were not observed in global network parameters between visually-cued oscillatory finger tapping and the resting condition; however, minor changes were noted in the degree and betweenness centrality in premotor and prefrontal regions in the gamma-band network. Thus, a fractal analysis of MEG/EEG signals as well as fMRI provides valuable insights into self-similarity involved in the complex systems of the human brain, and the sub-second dynamics of the fractal dimension following task execution warrant further studies.

### Self-organized criticality or the neuronal avalanche

Self-organized criticality is an interesting model in complex systems (Bak et al., 1987). In their early study, Bak and colleagues indicated that systems consisting of many elements with non-linear interactions self-organized into a state with statistical signs of critical systems, with spatial and temporal correlations of a power-law form being the statistical signs. The power-rule form is also called “scale-free” because it is the only mathematical function that indicates that the system has no typical or characteristic scale. The branching ratio is an important metric of criticality. There is a large amount of evidence to support self-organized criticality in non-human animal neurophysiology *in vitro* in terms of the branching ratio. Previous studies reported that the LFPs of spontaneous activity showed spatiotemporal cascades of discrete events, called the neuronal avalanche, in acute cortex slices, slice cultures, and the developing cortex of the anesthetized rat (Beggs and Plenz, 2003; Gireesh and Plenz, 2008; Lombardi et al., 2012; Yang et al., 2012; Shew and Plenz, 2013). This neuronal avalanche has also recently been detected in spontaneous LFPs and spike activity *in vivo* in sensory and motor-related regions (Petermann et al., 2009; Hahn et al., 2010). These cascades are characterized by a critical branching process. In branching processes, activity propagates from one active group of neurons to another in a cascade. Here, the ratio between activations in consecutive time steps is termed the branching ratio, σ. If the branching ratio is smaller than one, activity dies out before it has propagated far (sub-critical network), whereas a ratio larger than one produces an explosion of activity (super-critical network). When the ratio is around one, the system is critical and the activation of *N* neurons leads to the activation of other *N* neurons, operating at an exquisite balance at which activity propagates long distances without runaway excitation of the super-critical network. Various kinds of analyses of resting-state MEG signals have provided evidence for the presence of self-organized criticality by examining long-range temporal correlations and power-law scaling behavior (Linkenkaer-Hansen et al., 2001), the lifetime of the oscillatory avalanche (Poil et al., 2008), synchrony-based power-law scaling (Kitzbichler et al., 2009), and the size of the avalanche cascade (Shriki et al., 2013). Recent MEG and EEG studies reported a correlation between a long-range temporal correlation or avalanche dynamics and behavioral scaling laws (Palva et al., 2013; Smit et al., 2013) and developmental changes (Smit et al., 2011). Thus, the neuronal avalanche as a notable factor for identifying cortical dynamics at criticality has recently been suggested.

## Summary

This review focused on connectivity and network metrics derived from MEG/EEG signals in space, time, and frequency dimensions. Efficient analyses of MEG and EEG signals in the full data space may uncover hidden information embedded in these signals. The fusion of non-linear physical science and neuroscientific MEG/EEG studies has been characterizing the dynamic and complex systems of the human brain, and self-organized criticality and self-similarity are representatives. Therefore, this review shows that these sophisticated analysis methods of MEG/EEG signals hold tremendous potential for uncovering the various functions of the human brain in multidimensional space.

## Author contribution

TK, ET, and RK contributed to drafting and revising the paper.

### Conflict of interest statement

The authors declare that the research was conducted in the absence of any commercial or financial relationships that could be construed as a potential conflict of interest.
